# The Lateral Prefrontal Cortex and Selection/Inhibition in ADHD

**DOI:** 10.3389/fnhum.2018.00065

**Published:** 2018-02-20

**Authors:** Ziv Ronel

**Affiliations:** Hebrew University of Jerusalem, Jerusalem, Israel

**Keywords:** ADHD, inhibition, selection, control, LPFC

## Abstract

A previous paper from our lab (Shalom, 2009) presented evidence that the medial part of the prefrontal cortex is involved in the integration of raw, unintegrated information into coherent, wholistic mental representations such as perceptual objects, episodic memories, emotional states, and motor actions. It has used this analysis to classify some challenges encountered by people with Autism Spectrum Disorders, linking different types of difficulties in integration with different subareas of the medial prefrontal cortex. The current paper performs a similar analysis for the corresponding subareas of the lateral prefrontal cortex. It presents evidence that the lateral part of the prefrontal cortex is involved in the selection/inhibition of perceptual, memory, emotion, and motor aspects of processing. It then uses this analysis to classify challenges encountered by people with ADHD, linking different types of difficulties in selection/inhibition to different subareas of the lateral prefrontal cortex.

## Introduction

A previous paper from out lab (Shalom, 2009) argued that neurocognitive processing can be divided into three-levels: (1) a basic-level involving primary cognitive, emotional, and sensorimotor processing. For example, a loud unexpected sound that is perceived in primary auditory-sensory systems might trigger physiological emotional-responses, such as fear (fast heart beats, etc.). (2) an integrative-level, that combines the output of all primary processes from the basic-level, and forms a global-coherent meaning, experience, or behavior. For example, the mental representations of the various primary elements that constitute the fear emotional response results in a conscious feeling of being afraid. (3) A logical-level, which forms abstract logical rules (if-then rules) from the basic-level (e.g., “If I have fast heart beats and cold sweat, then I might be afraid”), and is also involved in selection and inhibition (e.g., controlling emotional urges). This three-level architecture was applied to four general psychological domains: emotion, memory, sensation-perception, and motor. Shalom (2009) focused on the second, integrative level and its relation to the four psychological domains. It also argued that these four types of integration are subserved by four different subareas of the medial prefrontal cortex: Brodmann Area (BA) 11 (perception), BA 10 (memory), BA 9 (emotion), and BA 8 (motor). Finally, it presented evidence that a selective deficit in these BA areas and these types of integrative processes underlie some of the common deficits in ASD (autism spectrum disorders).

**Figure 1:**
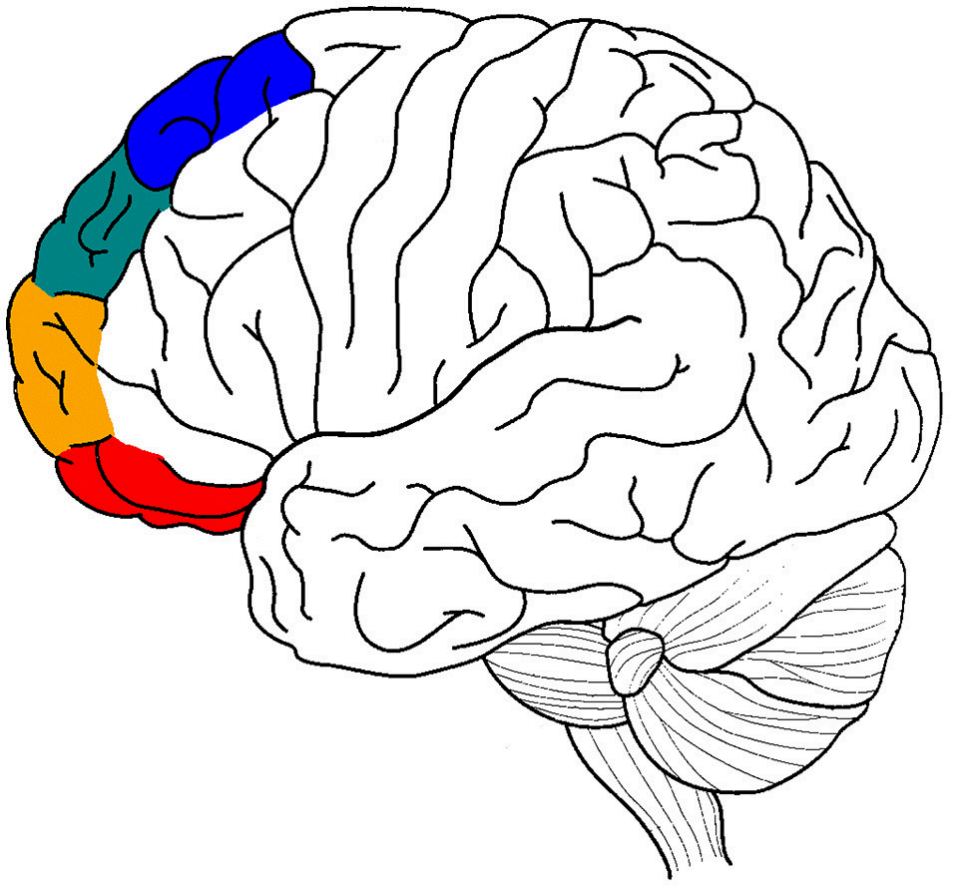
A graphic summary of the main anatomical hypotheses of the article. Motor processing in blue, emotion in green, memory in orange, and sensory-perceptual in red.

The current review attempts to perform a similar analysis for the lateral prefrontal cortex (LPFC), shifting from the relevant medial prefrontal areas to their lateral counterparts: lateral BA 11/BA 47 (perception), lateral BA 10/BA 46 (memory), lateral BA 9 (emotion), and lateral BA 8 (motor) (Figure 1), (cf. the distinction between clusters 1, 2, 3, and 4 of the anterior cingulate in Beckmann et al. (2009). It brings evidence that a selective atypicality in these BA areas and a selective deficit in selection/inhibition processes in these four cognitive domains is involved in some of the common deficits in ADHD (attention deficit hyperactivity disorder), such as difficulties inhibiting motor responses (e.g., inability to inhibit inappropriate movements), perceptual focus (e.g., inability to ignore distraction) and emotional reactions (e.g., inability to control urges) (Barkley, 1997; Nigg, 1999).

## Perception

### Perceptual selection and inhibition and lateral BA 11/47

There is evidence that perceptual selection and inhibition are supported by neural networks involving the lateral part of BA 11/47 in the LPFC. In a model proposed by Zsuga et al. (2016), the orbitofrontal cortex (OFC) is suggested to play a major role in the selection of visual stimuli according to task requirements.Specifically, they suggest that the medial part of the OFC plays a central part in integrating and categorizing information about stimuli from the environment and their context, while the lateral part of the OFC is involved in assigning and updating selection parameters according to task specific values of the available stimuli. In support of this claim, in one study, Howard and Kahnt (2017) showed that the lateral part of BA 11 is involved in encoding goal directed values of olfactory stimuli. They presented hungry participants with two preferred food smells and required them to choose whether to smell one or the other. Later, the participants were given one of the two foods to eat and were allowed to choose between the two smells once again. The change in preference toward the non-satiated smell was reflected by a change in activity in the lateral part of BA 11/47. Furthermore, it has been shown that lateral BA 11/47 is involved in the rejection of irrelevant stimuli (Kaufman et al., 2016).

Lateral BA 11/47 has been also shown to be involved in the maintenance of visual stimuli in working memory and guidance of visual selection. In one study, Soto et al. (2007), compared two conditions of a primed visual selection in which the prime was shown either once or twice. Lateral BA 11/47 activation was greater when the prime was shown twice, however, a reduction in activation was found when similar stimuli were passively repeated twice (without a visual selection phase, and therefore without task relevance).

Finally, according to Price (2007), lateral BA 11 is part of the sensory orbital network.

### ADHD and visual selection and inhibition

There is evidence that children and adults with ADHD have problems with inhibiting irrelevant visual and auditory stimuli. For example, ADHD adults have shown more interference effects (i.e., problems in inhibiting irrelevant stimuli) than controls when asked to complete a recall task while ignoring a background noise (Pelletier et al., 2016). Moreover, a positive correlation was found between task performance under conditions of irrelevant sound and the extent of attentional symptoms reported by patients on a clinical symptom scale. In another study, ADHD and control adults were asked to conduct a phone conversation while driving. The ADHD group showed significantly poorer driving skills during the phone conversation condition than in the silence condition when compared to controls (Reimer et al., 2010). Other studies have shown poor visual inhibition in ADHD individuals. For example, Forster et al. (2014) and Forster and Lavie (2016) found that ADHD adults performed a letter search task slower when irrelevant but salient cartoon characters were present on the screen, to a greater extent than controls. In a related manner, the Stroop interference task requires participants to attend a specific dimension of a visual stimuli while ignoring another. Studies found ADHD children to perform more poorly on the Stroop interference condition than controls (Sørensen et al., 2014), and that their performance correlated with their inattention and hyperactivity symptoms (Ikeda et al., 2013).

### ADHD, visual selection, and inhibition and lateral BA 11/47

In addition, several studies found specific evidence of atypical activation in lateral BA 11/47 in individuals with ADHD performing visual selection/inhibition tasks. Tsujimoto et al. (2013) asked children with and without ADHD to perform a working memory task with and without distraction. The ADHD participants showed significantly poorer behavioral performance, particularly under distraction, and showed significantly higher levels of activation in lateral BA 11/47 than controls. In a different study, Yasumura et al. (2014) tested ADHD and control children on both Stroop interference and reverse Stroop interference conditions, and found that in both conditions ADHD children performed more poorly and that performance correlated with lateral BA 47 activity. In another study where a salient yet irrelevant distractor was presented during a time estimation task, it was found that while the distractor assisted the ADHD participants to the same degree as the controls, the lateral BA 11 activation which accompanied the distractor's appearance was significantly larger (Pretus et al., 2016).

## Memory

### Memory selection and inhibition and lateral BA 10/46

While it is not a general consensus that BA10/46 is engaged in episodic memory functions, there is much evidence to support a role for BA 10/46 in the selection and inhibition of memory information. For example, Kim (2011), analyzed 74 studies that compared remembering and forgetting (i.e., successfully remembered items vs. distractors judged erroneously to have appeared), and found that the greatest bulk of studies overlapped at the frontal part of BA 46, near it's junction with lateral BA 10. In a later study, Kim (2013) performed a meta-analysis of 70 studies focusing on new and old items (i.e., hits vs. correct rejections). Results show that new>old comparisons yielded activations that spread across the middle part of the left lateral prefrontal. However, when focusing on source memory studies in which participants are required to retrieve not only the target item but also an additional detail related to the item from the learning phase (i.e., when selection demands are higher), specific activation was seen in the frontal part of BA46 including the rostral part of BA10.

A different line of evidence comes from a meta-analysis by Gilbert et al. (2006), in which 104 studies were analyzed focusing on BA 10, and found that the majority of studies involving the lateral part of BA 10 tested working memory and episodic memory retrieval, while studies involving the medial part of BA 10 tested mentalizing (i.e., attending to one's own emotions and mental states or those of other agents).

In addition, Cohen et al. (2014), employed the value directed remembering task, in which participants are presented with word lists in which some words are assigned more value than others, thus encouraging participants to attempt and select higher value items over low value ones during encoding. Results show specific activation of right lateral BA46/10, and very pronounced activation of left lateral BA46/10, when a cue predicting a high value word was appearing.

### ADHD and memory selection and inhibition

There are several studies that have found evidence for difficulties in the selection and inhibition of memory information in ADHD. For example, in a study by Pollak et al. (2007), adults with and without ADHD performed a difficult list learning task in which they had to memorize and recall five word lists on eight different occasions. Results show that the ADHD participants made more double recalls and intrusion errors (recalling words from the wrong list), which are examples of a difficulty in inhibiting the retrieval of task irrelevant items. In a similar study, Soliman and Elfar (2017), asked adults with and without ADHD to learn eight lists and were then presented with a recognition task. The ADHD group recognized less words correctly, produced more false positive responses (i.e., wrongfully selecting items that weren't learned) and were more confident in their mistakes. In a different study, Castel et al. (2011), asked children with and without ADHD to perform the value directed remembering paradigm mentioned above. Results show that children with ADHD recalled as many items as the controls, however they were less able to select which items to remember.

### ADHD, memory selection, and inhibition and lateral BA 10/46

The literature search yielded only one neuroimaging study assessing selection or inhibition of memory functions in ADHD. In a study by Depue et al. (2010), adults with and without ADHD were taught a relation between face-picture pairs until mastery. They were later shown a picture of a face and were asked to either think of the related picture or to keep the related image from coming into consciousness. Results show that ADHD participants activated lateral BA 10 less than controls when comparing the “no-think” condition with the “think” condition.

## Emotion

### Emotion selection and inhibition and lateral BA 9

There is much evidence supporting the relation between emotion regulation (the inhibition of certain emotions and selection of others) and lateral BA 9. For example, in a study by Hallam et al. (2014), participants viewed emotion inducing films and were required to either suppress or reappraise their emotional reaction, or to simply watch the film. Results showed that both reappraisal and suppression compared to the passive condition showed activation of lateral BA 9. Similarly, two studies compared reappraisal and passive watching of pictures in people with PTSD and controls. Both studies found lateral BA 9 to be activated more during the reappraisal condition, but to a lesser extent in people with PTSD (Xiong et al., 2013; Rabinak et al., 2014).

In addition, two different meta-analyses were used to examine fMRI studies assessing emotion regulation. Buhle et al. (2014) found that reappraisal involving down regulation of negative affect consistently activated lateral BA 9; Frank et al. (2014) found that such reappraisal was accompanied by greater activation in lateral BA 9 and decreased activation in the amygdala.

### ADHD and emotion selection and inhibition

Several studies found emotion regulation deficits in children with ADHD. For example, several studies show that children with ADHD have a harder time suppressing their emotions than their typically developing (TD) peers. For example, in one study children underwent a peer competition task, in which half were requested to hide their emotions and half were not. According to assessments made by naive judges, results show that ADHD children were less able to mask their emotions than their peers (Walcott and Landau, 2004). Furthermore, a study assessing the performance of children with ADHD on an emotional Stroop task, found that the children with ADHD had a harder time inhibiting responses to angry and frustrated faces (Yarmolovsky et al., 2016).

Additionally, in a meta-analysis of emotion regulation in ADHD performed by Graziano and Garcia (2016), they found four domains of interest, namely: recognition (i.e., ability to process and infer the emotions of others as well as one's self), reactivity (i.e., the threshold, intensity, and duration of one's affective arousal), regulation (i.e., effectively responding to emotional reactivity in a flexible manner that facilitates adaptive functioning), and empathy (i.e., the ability to experience another's affective state and/or express concern for another's position). Results show that while all four domains seem to be atypical in the ADHD population, the most notable deficiencies occur in the reaction and regulation domains. This means that (especially) children with ADHD tend to react more quickly, more intensely and for a longer period of time to aversive situations, and are less able to regulate these emotions even when they attempt to do so.

### ADHD, emotion selection, and inhibition and lateral BA 9

There are several studies that show specific atypicalities in lateral BA 9 activation in ADHD participants performing tasks which require emotion regulation. For example, in a study by Passarotti et al. (2010a), children with bipolar disorder, children with ADHD and a group of age matched controls performed an emotional valence Stroop task. In this task, positive, neutral and negative words were presented, and participants were required to respond according to the word's color while ignoring its meaning. Results show that the bipolar group had difficulty ignoring positive words, while the ADHD group had difficulty ignoring negative words. Interestingly, lateral BA 9 activation was more pronounced for task condition that was successfully performed, i.e., for negative words in the bipolar group, and for positive words in the ADHD group. Furthermore, in a Stroop based emotional task, Hwang et al. (2015), asked children with and without ADHD to perform a numeric Stroop task after being shown pictures that were either positive, neutral or negative. While behavioral data did not show significant differences between the different emotional conditions, they did find that children with ADHD were less able to recruit lateral BA 9 when performing the task, a trait which also correlated with symptom severity as was measured by the Conner's parent scale.

## Motor

### Motor selection and inhibition and lateral BA 8

There is much evidence that the inhibition of motor actions is supported by neural networks in lateral BA 8. The most common measure of motor inhibition is the Stop Signal Task (SST) in which participants are required to press a key when a stimulus is presented, but refrain from executing that key press if the stimulus is followed shortly by a signal. Studies show that inhibiting the key press involves activation in lateral BA 8 (Matthews et al., 2005; Smith et al., 2013; Hughes et al., 2014). Lateral BA 8 has also been shown to be specifically active when the selection of a motor response required attention or involved conflict. For example, Enriquez-Geppert et al. (2013), asked participants to press a key when a stimulus appeared on the screen, however, the response key changed in some of the trials. The Experimenters found lateral BA 8 to be specifically activated on trials in which the response key was different. Similarly, Lenartowicz et al. (2011), used a go/no-go task in which after a few practice sessions, the stimuli changed so that the previous go signal became a no-go signal (requiring more inhibition). They found lateral BA 8 to be specifically activated on those trials. In a similar study Albares et al. (2014), used a version of a go/no-go task in which a prime was presented before the go and no-go stimuli. However, while a green light indicated that a go stimulus will follow, a red light meant that any one of three conditions may occur, either a go stimulus, a no-go stimulus or no stimulus at all. The researchers reasoned that the red-light condition involves more complex motor planning than the green light, and found that lateral BA 8 was indeed more active during that condition.

### ADHD and motor selection and inhibition

There is robust evidence of motor selection and inhibition deficits in children with ADHD. Many studies to date have shown that children, adolescents and adults with ADHD show poorer performance on different versions of the stop signal task (e.g., Rubia et al., 1998; Lee et al., 2016; Bialystok et al., 2017; Dekkers et al., 2017), and that when children with ADHD are properly medicated and motivated using effective reinforcement, these differences may disappear (Rosch et al., 2016). Furthermore, in a very large study by Crosbie et al. (2013), ADHD symptoms were measured in over 16,000 general public children who performed the SST. Results showed that individuals with greater ADHD trait scores had worse response inhibition, slower response latency and greater variability, and also that this trend was highly heritable. Similarly, Alderson et al. (2007), performed a meta-analysis focusing on ADHD children and the SST and found greater mean response times, greater response variability and greater stop signal response time in the ADHD group.

### ADHD, motor selection, and inhibition and lateral BA 8

There is also considerable evidence showing specific atypicalities in lateral BA 8 activation in ADHD participants performing tasks which require motor selection and inhibition. For example, two separate meta-analyses have explored fMRI studies assessing motor inhibition in children and adults with ADHD (Hart et al., 2013; Rubia et al., 2013). Both meta-analyses found lower activation in lateral BA 8 to be related to motor inhibition measures, and the study by Rubia et al. (2013), also found methylphenidate to increase activation in lateral BA 8 which was related to improved performance on motor tasks. In addition, studies which employed different versions of the SST and the go/no-go task found less activation in lateral BA 8 in the ADHD group during a simple no-go condition (Passarotti et al., 2010b; Mulligan et al., 2011), moments before an error occurred (Spinelli et al., 2011), as well as during a free choice condition in which participants chose whether to press a button or not when a stimulus was presented (Karch et al., 2010).

## Summary

The current paper presents evidence that the lateral part of the prefrontal cortex is involved in the inhibition, selection and manipulation of motor, emotional, memory, and perceptual-sensory information, a function that is helpful in everyday life through the ability to ignore distraction, select and retrieve specific information from our memories, identify and regulate our emotional state or plan a situation appropriate motor response. It uses this model to classify challenges encountered by people with ADHD, linking different types of difficulties in selection/inhibition to different subareas of the lateral prefrontal cortex.

## Author contributions

The author confirms being the sole contributor of this work and approved it for publication.

### Conflict of interest statement

The author declares that the research was conducted in the absence of any commercial or financial relationships that could be construed as a potential conflict of interest.
